# Editorial: The Future of Work in Non-profit and Religious Organizations: Current and Future Perspectives and Concerns

**DOI:** 10.3389/fpsyg.2020.623036

**Published:** 2020-12-08

**Authors:** Antonio Ariza-Montes, Gabriele Giorgi, Horacio Molina-Sánchez, Javier Fiz Pérez

**Affiliations:** ^1^Social Matters Research Group, Universidad Loyola Andalucía, Seville, Spain; ^2^Department of Human Sciences, European University of Rome, Rome, Italy; ^3^Department of Financial Economics and Accounting, Universidad Loyola Andalucía, Seville, Spain

**Keywords:** non-profits and religious organizations, employment, sustainable work, employees commitment, well-being

Non-profits and religious organizations support the state's public services contributing to mitigate critical situations in developing countries, or to improving the welfare status in developed countries. Despite their unquestionable social role, little is known about the functioning of these organizations from a management point of view. In the past, the survival of these entities has been based on the identification and commitment displayed by some of their members: partners, donors, volunteers or members of religious associations, among others.

Recent studies have shown that working in such organizations is very demanding from an emotional point of view, to this we must add workers' low salaries, the instability of the job position and the extenuating work time. All of this leads to low levels of well-being and higher staff turnover rates.

The decreased admission of members in religious organizations together with the reduced capacity of the non-profit organizations to attract and maintain the employees might jeopardize the long-term survival of these entities. Despite this risk, there is a marked resistance to change in the sector that embodies, among other things, an aversion to anything that involves the incorporation of criteria concerning professional management. Indeed, it is believed that professionalism would create a change of style exclusively focused on increasing the productivity, leading, in worst cases, employees to question the principles motivating them to work in a non-profit or religious organization.

The challenge in this situation is for professionalization to be respectful toward the mission and charisma moreover to present an opportunity for the employees' personal growth. Furthermore, technological and digital improvements are expected due to the recent deployment of machines, robots, digital connectivity, and artificial intelligence in the workplace. Non-profit and religious organizations may suffer from technological development; the risk is to decrease the human side, and orientation characterizing these organizations.

In this context, the increasing rate of non-standard forms of employment has replaced the traditional productive organization, based on full-time and permanent employment, with a system based on flexible under-employment, plural, and decentralized work causing instability, non-protection and vulnerability of the employee. For example, these concerns are presented by the International Labor Organization's Future of Work Initiative, that calls for an in-depth examination of the future of work to provide decent and sustainable work opportunities for everyone. Promoting a sustainable and decent future of work is essential to focus on research to promote higher levels of work and society, decent jobs for employees, work for the organization and work production, and finally organized governance of work.

Accurately, this special issue is focused on the future of work in non-profit and religious organizations. Most of the 14 articles published in this special issue are empirical contributions involving 54 authors from different regions of the world, mainly from Europe and America. The published works approximate a wide range of entities (charitable organizations, non-profit hospitals, fraternities and brotherhoods, catholic schools.), addressing the topic of research from different areas and fields of knowledge, such as Management, Economics, Occupational & Organizational Psychology, law, Humanities and Philosophy, Sociology and Social Work and Ethics and Sustainability, among others.

The study of the same phenomenon from different prisms offers a variety of perspectives that undoubtedly enrich scientific knowledge on the subject. In this sense, the contributions of this special issue make an interdisciplinary approach that will improve the overall well-being of workers, their effectiveness, commitment and productivity. All this will help ensure The Future of Work in Non-Profit and Religious Organizations.

As a whole, the published manuscripts address four significant areas of interest to the academy. First, five articles focus on the performance of such organizations in order to ensure the long-term survival of such organizations. Descending to the micro-level, the second area of interest is the non-profit organization-client relationship, whether it is an immigrant, patient, student or any other beneficiary of the service provided by these organizations in society. Specifically, three articles highlight the importance of proactively managing this relationship as a critical factor in the success of these organizations in fulfilling their mission. Without moving away from the micro-level, the third area of interest is the different groups that collaborate in this type of entity (lay, religious, volunteer, employees.), analyzing different outcome variables that show what it means for them to work (in exchange for economic remuneration or without it) in an entity that puts social purposes before profit. The fourth and final area of interest is a call of attention to the managers of these entities, warning them of the need to exercise strong and committed leadership. In this sense, two of the published articles analyze the style of leadership in these organizations, betting on servant leadership as the most consistent with the mission of these entities.

## Improving Performance in Non-Profit and Religious Organizations

As noted above, five manuscripts treat the performance of the non-profit and religious organization.

The article by Acosta-Prado et al. uses a health sector personnel working in Colombian non-profit hospitals (NPHs) to analyze certain human resources practices that can reinforce any regular organizational performance. Results obtained through Partial Least Squares Structural Equation Modeling (PLS-SEM) confirm the influence of human resource management on innovative performance, representing an excellent opportunity for NPHs.

The qualitative study conducted by López-Cabrera et al. analyzed four types of conflicts (task, process, status, and relationship conflicts) in a large non-profit organization. From the perspective of organizational psychology, the most relevant finding obtained by these authors is the significant difference between paid staff and volunteers in conflict perceptions, showing paid staff, overall, higher levels of conflicts than volunteers. Findings also show more substantial negative consequences for paid staff compared to volunteers.

The paper by Retolaza et al. discusses the use of social accounting to calculate the social value generated by religious-oriented organizations. Analyzing 16 educative centers of the diocese of Bilbao (Spain), the authors highlighted the utility of social accounting in three significant fields: (1) external communication mainly to the Basque government (who is the primary provider of funding for the centers) and concerning other stakeholders; (2) information for management and strategic planning; and (3) empowerment of stakeholders, specially collaborators.

In the fourth article, Buenadicha-Mateos et al. point out the importance of attracting and retaining the best employees in an increasingly competitive context, a topic that is still scarce in the voluntary sector. Thus, online recruitment is a growing trend. This study sheds light on the topic because it examines and compares the 100 best companies to work for, published by Fortune, and the 100 largest charities, reported by Forbes. The research of these authors concludes that benchmarking efforts can help increase the charities' attractiveness in the labor market shortly.

In a similar vein, the use of digital platforms in order to attract the most well-prepared and motivated young volunteers into the projects of non-profit organizations is the main objective of the fifth article. Saura et al. adopt the extended Technology Acceptance Model (TAM) to evaluate data from a sample of potential volunteers from non-profit organizations. Using a structural equation approach, they confirm that extended TAM is an appropriate model for the analysis of technological acceptance of social platforms that allow volunteers or other professionals to be recruited.

## Organization-Customer Relationship

Three studies explore the client-organization relationship as a critical factor in success in non-profit entities.

First, Foxall et al. highlight differences in managerial work between business firms and non-profits exemplified by the charitable organization. Adopting as a frame of reference the theory of the marketing firm, researchers describes the essentials of the theory and its basis in consumer behavior analysis and outlines its unique position as the organization responsible for marketing transactions, based on objective exchange, competitive markets and prices, and the deployment of the entire marketing mix. Moreover, they discuss how the marketing firm differs from charities in terms of goal separation, market-based pricing and competition, the entrepreneurial (strategic) process, the pursuit of customer-oriented management, and organizational structure.

Second, Sánchez-Hernández et al. use a health religious organizations sample to focus their attention on the nurse-patient relationship and empirically explore a theoretical model that links nurses' suffering at work with personal's willingness to engage in a therapeutic and spiritual relationship with patients and the consequent effect on quality. One hundred two nurses were investigated through an analytical case-study based on Partial Least Squares (PLS) path modeling. The main conclusion of this research is the necessary inclusion of suffering, even in good places to work, as a critical indicator for better management, which highlights the need to implement better human management systems in these organizations.

Finally, Robina-Ramírez et al. obtain a sample of 214 biology and religion teachers from 118 Catholic schools from which they analyze the critical role that teachers play in their relationship with students in this type of center. In particular, they focus on the environmental competencies of teachers in order to investigate the environmental attributes that should be incorporated into religious schools to combine religious and environmental teaching. In line with the experiences of international programs for eco-schools, the results of this research confirm that environmental competencies must be developed in teachers to enable the greening of schools.

## The Human Factor in Non-Profit and Religious Organizations

Up to four articles focus on different outcome variables related to the working conditions and well-being of people collaborating with these entities.

From the Malmo University of Sweden, Edvik et al. discuss the importance of work engagement and job satisfaction as an organizational response to changes in the environment. Using as a frame of reference the job demands and job resources model, and as a working methodology the regression analysis in a sample of 2,112 employees of the Church of Sweden, these authors demonstrate that employees' credence in the organization's ability to respond to change is critical to consider for understanding employee well-within being religious organizations. In conclusion, the study suggests that organizations that are built upon strong values and institutionalized beliefs need to tread carefully in the process of adapting to conformal pressure for change. This, since the actions and choices of the organization, are important for employees' credence in the organization and, in turn, employee well-being.

Work engagement is also one of the principal research objects of Ortiz-Gómez et al.'s work. These authors stress the importance that it represents for religious organizations to serve the community while transmitting specific values according to the charism of the order. Using Partial Least Squares method, in a sample of nearly a thousand workers from a Catholic religious organization, the results of the study confirm that there is a direct relationship between the human values of the members, the level of authenticity of the members and their commitment to the organization, as well as a mediating effect of authenticity on the relationship between human engagement values and work.

Landing in the international boundary between the United States and Mexico, and building on theories of Planned Behavior and Reasoned Action, Suárez et al. investigate the relevance of volunteer commitment as an instrument for managing cross-border conflicts in the particular context of San Diego and Tijuana Area. These authors suggest that intentions to cooperate with non-government institutions are influenced directly by attitudinal values and indirectly by their beliefs related to social conflicts. By using a qualitative methodology based on interview data, Suárez et al. provide new insights into how organizational and relational elements impact sustainable volunteer management and point out the role played by attitudes toward non-government institutions demonstrating the relevance of volunteer commitment, transforming part of the positive toward attitude social problems into a significant intention to cooperation.

If voluntary activities enhance the integration of older people into society, their participation will help to generate economic resources and improve their welfare. It is the starting thesis of Gil-Lacruz et al.'s article. These authors analyze the well-being generated in older people by carrying out volunteering activities. To achieve their goals, they develop an empirical study using micro-data from the World Value Survey and macro-data from the statistics of the OECD. The results of his research suggest, on the one hand, that volunteering might improve every indicator of well-being except happiness, and on the other hand, that the impact on individual well-being depends on the type of volunteering activity that develops.

## Leading People in Non-Profit and Religious Organizations

The special issue also includes two articles that emphasize the importance of leading very heterogeneous groups to bring their full potential to the service of the organization.

Professors Hernández-Perlines and Araya-Castillo contribution analyses the relationship between servant leadership, innovative capacity and performance in a sample of Third Sector entities. Using a dual methodology, both quantitative (second-order structural equations, PLS-SEM) and qualitative comparative analysis (QCA), the authors of this article conclude that the servant leadership is the most consistent for the philosophy of such organizations, positively influencing the performance of these entities.

The religious fraternities and brotherhoods of Seville, Spain, are among the principal agents of the social aid carried out in that city. It is the research object of Blanco et al.'s work. These authors aimed to verify, through a revised version of the Rivera and Santos questionnaire (2015), if the leader-server characteristics, usually apply to a natural person, can also be identified in such corporations. In addition to this methodological objective, the resulting product was used to identify the shared characteristics that make the fraternities and brotherhoods of Seville different from other private agents fighting against poverty.

## Conclusion

In conclusion, it should be noted that the work carried out by 54 authors who have worked on a joint sample of thousands of members has highlighted the need for proactive management of non-profit and religious organizations. These entities are based on service leadership and differentiated according to the particularities, desires, needs and expectations of the different groups of people working for the effectiveness and efficiency of these entities. If this is joined by an effort to measure the value these organizations bring to society, which will undoubtedly make them gain in transparency and legitimacy, the long-term survival of the non-profit and religious organization is guaranteed.

## Author Contributions

All authors listed have made a substantial, direct and intellectual contribution to the work, and approved it for publication.

## Conflict of Interest

The authors declare that the research was conducted in the absence of any commercial or financial relationships that could be construed as a potential conflict of interest.

